# The Classification of Six Common Skin Diseases Based on Xiangya-Derm: Development of a Chinese Database for Artificial Intelligence

**DOI:** 10.2196/26025

**Published:** 2021-09-21

**Authors:** Kai Huang, Zixi Jiang, Yixin Li, Zhe Wu, Xian Wu, Wu Zhu, Mingliang Chen, Yu Zhang, Ke Zuo, Yi Li, Nianzhou Yu, Siliang Liu, Xing Huang, Juan Su, Mingzhu Yin, Buyue Qian, Xianggui Wang, Xiang Chen, Shuang Zhao

**Affiliations:** 1 Department of Dermatology Xiangya Hospital Central South University Changsha China; 2 Hunan Engineering Research Center of Skin Health and Disease Xiangya Hospital Central South University Changsha China; 3 Hunan Key Laboratory of Skin Cancer and Psoriasis Xiangya Hospital Central South University Changsha China; 4 National Clinical Research Center of Geriatric Disorders Xiangya Hospital Central South University Changsha China; 5 Xiangya School of Medicine Central South University Changsha China; 6 Tencent Medical AI Lab Shenzhen China; 7 Day Surgery Center Xiangya Hospital Central South University Changsha China; 8 Department of Computer Science National University of Defense Technology Changsha China; 9 School of Automation Central South University Changsha China; 10 Department of Electronic Information Engineering Xi’an Jiaotong University Xi'an China; 11 Department of Ophthalmology, Xiangya Hospital, Central South University Changsha China

**Keywords:** artificial intelligence, skin disease, convolutional neural network, medical image processing, automatic auxiliary diagnoses, dermatology, skin, classification, China

## Abstract

**Background:**

Skin and subcutaneous disease is the fourth-leading cause of the nonfatal disease burden worldwide and constitutes one of the most common burdens in primary care. However, there is a severe lack of dermatologists, particularly in rural Chinese areas. Furthermore, although artificial intelligence (AI) tools can assist in diagnosing skin disorders from images, the database for the Chinese population is limited.

**Objective:**

This study aims to establish a database for AI based on the Chinese population and presents an initial study on six common skin diseases.

**Methods:**

Each image was captured with either a digital camera or a smartphone, verified by at least three experienced dermatologists and corresponding pathology information, and finally added to the Xiangya-Derm database. Based on this database, we conducted AI-assisted classification research on six common skin diseases and then proposed a network called Xy-SkinNet. Xy-SkinNet applies a two-step strategy to identify skin diseases. First, given an input image, we segmented the regions of the skin lesion. Second, we introduced an information fusion block to combine the output of all segmented regions. We compared the performance with 31 dermatologists of varied experiences.

**Results:**

Xiangya-Derm, as a new database that consists of over 150,000 clinical images of 571 different skin diseases in the Chinese population, is the largest and most diverse dermatological data set of the Chinese population. The AI-based six-category classification achieved a top 3 accuracy of 84.77%, which exceeded the average accuracy of dermatologists (78.15%).

**Conclusions:**

Xiangya-Derm, the largest database for the Chinese population, was created. The classification of six common skin conditions was conducted based on Xiangya-Derm to lay a foundation for product research.

## Introduction

Skin conditions affect 1.9 billion people [1] and are the fourth-leading cause of the nonfatal disease burden across 188 low- and high-income countries [2-4]. Patients with skin diseases usually account for 10% of the number of outpatients in Chinese general hospitals and are also among the most common patients in primary care. However, there is a severe lack of dermatologists, particularly in rural Chinese areas. Based on incomplete statistics from the Chinese Society of Dermatology and Sexually Transmitted Diseases, there are approximately 30,000 dermatologists in China, accounting for 0.0016% of the total number of people with skin diseases.

The total number of dermatologists in China is less than 30,000, which results in a large gap between the supply and demand of dermatological resources, especially in primary care hospitals. In addition, some long-standing problems in the Chinese medical industry still exist, such as the uneven distribution of high-quality doctor resources, primary care hospital doctors prone to misdiagnosing patients, missed diagnoses, and insufficient knowledge of rare diseases [5,6]. Addressing these problems necessitates the use of artificial intelligence (AI) in Chinese dermatology. AI has transformed health care [7] and has become one of the most trending research topics. Deep learning algorithms, powered by advances in computation and the creation of large databases, have recently been shown to outperform humans in visual tasks [8]. Recent studies in the medical field, such as those on automatic lesion segmentation and intelligent disease recognition, have successfully used AI to identify skin diseases based on image data [9]. The use of AI in Chinese dermatology started later than in some high-income countries but has been developing rapidly; it could be a solution to the problems that exist in dermatology.

Using deep learning methods for skin disease identification requires a large amount of data. Skin images from the Chinese population are limited. Most public databases on skin diseases are from the US or European Union (EU) population, with a relatively small size and without corresponding pathology information to support their validity [9,10]. The images in the ISIC database, for example, come mainly from light-skinned populations in the United States, Europe, and Australia [11]. Other researchers prefer to use data sets established based on their own patients. These data sets contain information about limited types of skin diseases, include a smaller sample size, or have decreased reliability [12,13]. These problems hinder the use of Chinese AI products, because the systems developed based on these data sets are unreliable for automatically diagnosing members of the Chinese population. Therefore, it is necessary to build a data set with skin images from the Chinese population that would enable successful diagnosis of skin diseases for Chinese patients.

In this paper, we aim to build a database consisting of skin images based on the Chinese population and present an AI-based method for classifying six common skin diseases to lay a foundation for further product research.

## Methods

### Construction of Xiangya-Derm

As approved by the ethics committee of Central South University, we captured each image with either a digital camera or a smartphone in the Dermatology Outpatient Department, which relies on the Xiangya Big Data Collection Platform. The images were collected according to uniform standards from many cooperating hospitals in Nanjing, Guangzhou, Wuhan, Changsha (cities in China), etc. Most of these images have corresponding pathology information to ensure the accuracy of diagnosis information. For the other common and easy-to-diagnose skin diseases, images were verified by at least three experienced dermatologists. Meanwhile, the dermatologists were asked to annotate the specific location of the skin lesions in each image using an open source software called LabelImg [14]. Finally, the images with a clear diagnosis were added to Xiangya-Derm, a Chinese database. We classified the existing data into six types of skin diseases (malignant tumor, benign tumor, erythema papule scale, bullous, allergic, and connective tissue) based on the classification of common skin diseases and the amount of existing data.

### Study of Six Common Skin Diseases

After the construction of Xiangya-Derm, we conducted research on the classification of some common skin diseases from the Xiangya-Derm database using a convolutional neural network (CNN). First, six different subtypes of common dermatoses with additional data magnitudes and common outpatients (psoriasis, basal cell carcinoma, seborrheic keratosis, pemphigus, eczema, and lupus erythematosus) were selected, and a data set consisting of 5660 clinical images was constructed. These six subtypes of skin diseases were selected because they are the most common and representative skin diseases in primary care hospitals in China and because more data were available for these diseases than for any other. This data set contained common skin disease types, such as skin tumors, erythema, and scaling disease. In this data set, the image numbers of psoriasis and other diseases were unbalanced. If the data set had been used directly to classify each type of disease separately, it would have created a severe bias in the network against psoriasis. Therefore, we downsampled psoriasis samples to balance the number of samples among different categories. Moreover, we re-established a database containing six types of diseases, totaling approximately 3000 images, which ensured that the number of samples in different categories was within a reasonable range. In this range, the disease with the largest number of samples had over 1000 images, while the disease with the fewest samples had fewer than 200 images.

Based on a balanced number of samples in different categories, we proposed a network, called Xy-SkinNet, that was trained to be a six-category model for these six common types of diseases. Before we constructed the model, images in the database for modeling were checked. We found that the background of the clinical images in our database was complex and the size of lesions was relatively small. To overcome these problems and improve the classification performance, the model was designed to first detect the regions of interest that contained lesions. A specially designed information fusion block was then used to output the result by considering all the regions. The network used modified ResNet (M-ResNET) as the backbone network and Faster R-CNN [15], a two-stage object detection method, as the basic structure. Regarding the training and testing data set size, we trained our model with 2400 images and tested it with the remaining 600 images (training size:testing size=4:1) in the database.

**Figure 1:**
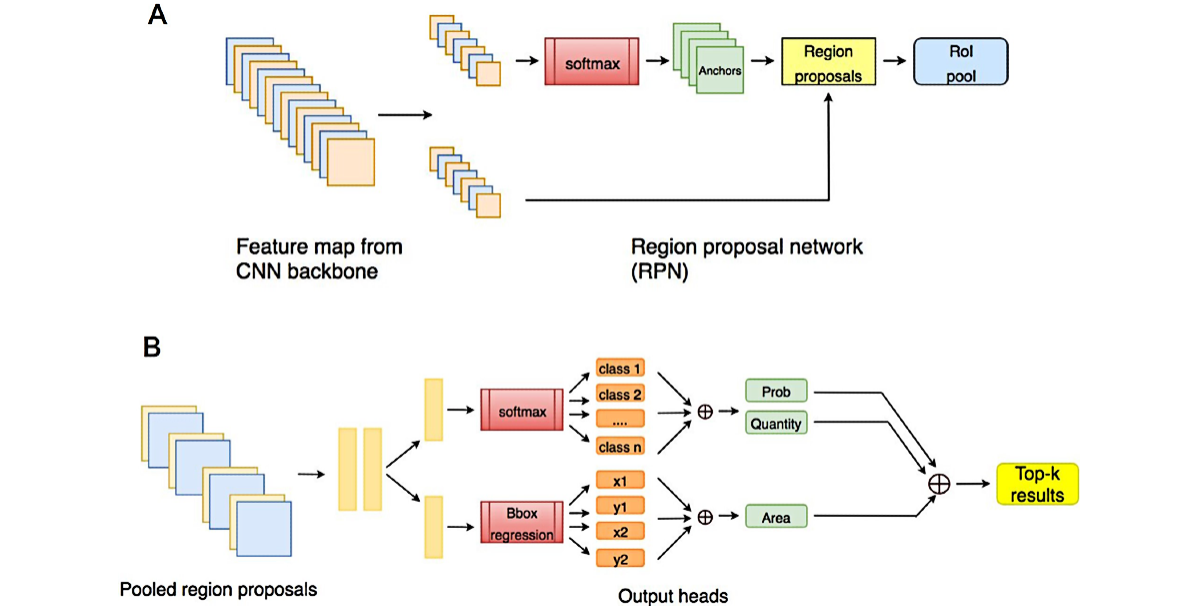
Structure of Xy-SkinNet. CNN: convolutional neural network.

Faster R-CNN is an improved version of R-CNN [16] and Fast R-CNN [17]. As a two-stage method, Faster R-CNN comprises two parts. The first part is a region proposal network (RPN), which generates several candidate regions on the entire image according to some preset box (anchor) and keeps only the foreground regions. The second part classifies and locates each remaining region in a fine-grained manner. These two parts share some network parameters. The structure of the model is shown in Figure 1. Based on the basic structure of the method, we improved it by adding an information fusion block for dermatosis characteristics.

### Human Versus Computer Competition

To evaluate the performance of Xy-SkinNet and validate its effectiveness in a clinical situation, we conducted a contrast experiment with 31 dermatologists of different seniority levels, including professors, senior attending doctors, young attending doctors, and medical students of dermatology. In our contrast experiment, Xy-SkinNet and all dermatologists had to identify the type of disease from a set of 100 clinical images without a time limit. Their performances were evaluated using accuracy as the metric. To ensure the fairness and validity of the comparison, all the images used contained only one type of skin disease and had been previously verified using corresponding histopathological information. Histopathological examination was the golden standard for diagnoses of these six types of skin diseases. Compared with other diagnostic technologies, histopathological examination was more accurate and objective. If there were some cases whose histopathological features were atypical, we let another three specialists (who did not take part in human vs. computer competition) determine a final diagnosis. Images for this contrast experiment were selected by dermatologists who did not participate in the experiment, and the images were not included in the training or testing data sets used in the process of building of our model.

## Results

### Xiangya-Derm, a Chinese Dermatological Database

**Figure 2:**
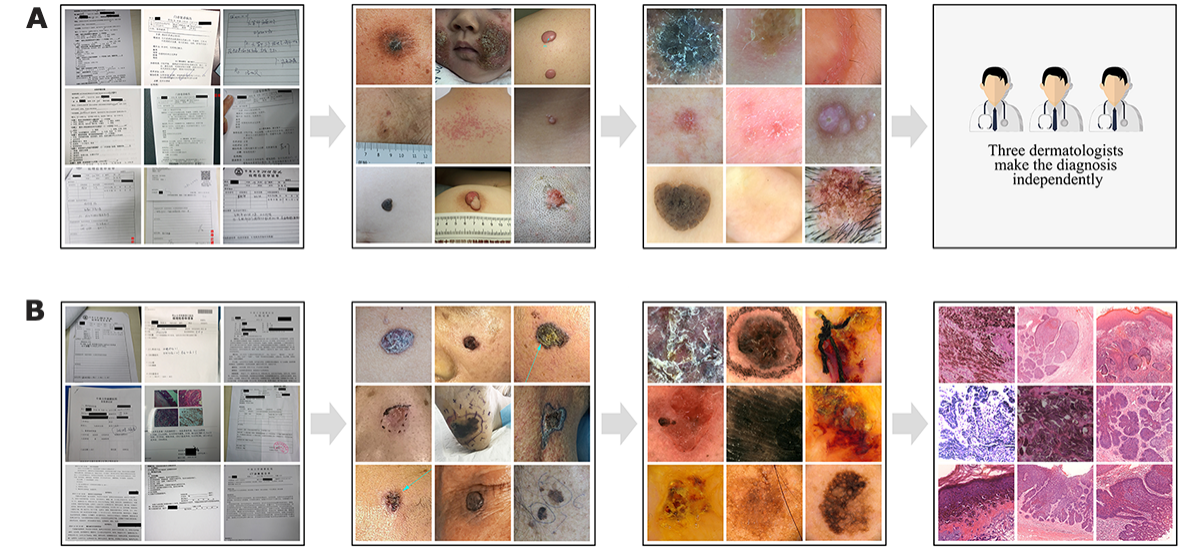
Pattern of the Xiangya Big Data Collection Platform.

Xiangya-Derm, as a new Chinese database, contains nearly 20 years of data from 15 hospitals, including Xiangya Hospital Central South University, the Third Hospital of Central South University, and Henan Provincial People’s Hospital. Most images were matched with pathology information to ensure the accuracy of the image diagnostic information. Other common skin diseases that are easily recognized by the naked eye were independently confirmed by three dermatologists. Each patient’s clinical history was recorded on the Xiangya Big Data Collection Platform. It is worth mentioning that most of the data in the Xiangya-Derm database have been labeled with bounding boxes by skin experts (Figure 2).

**Figure 3:**
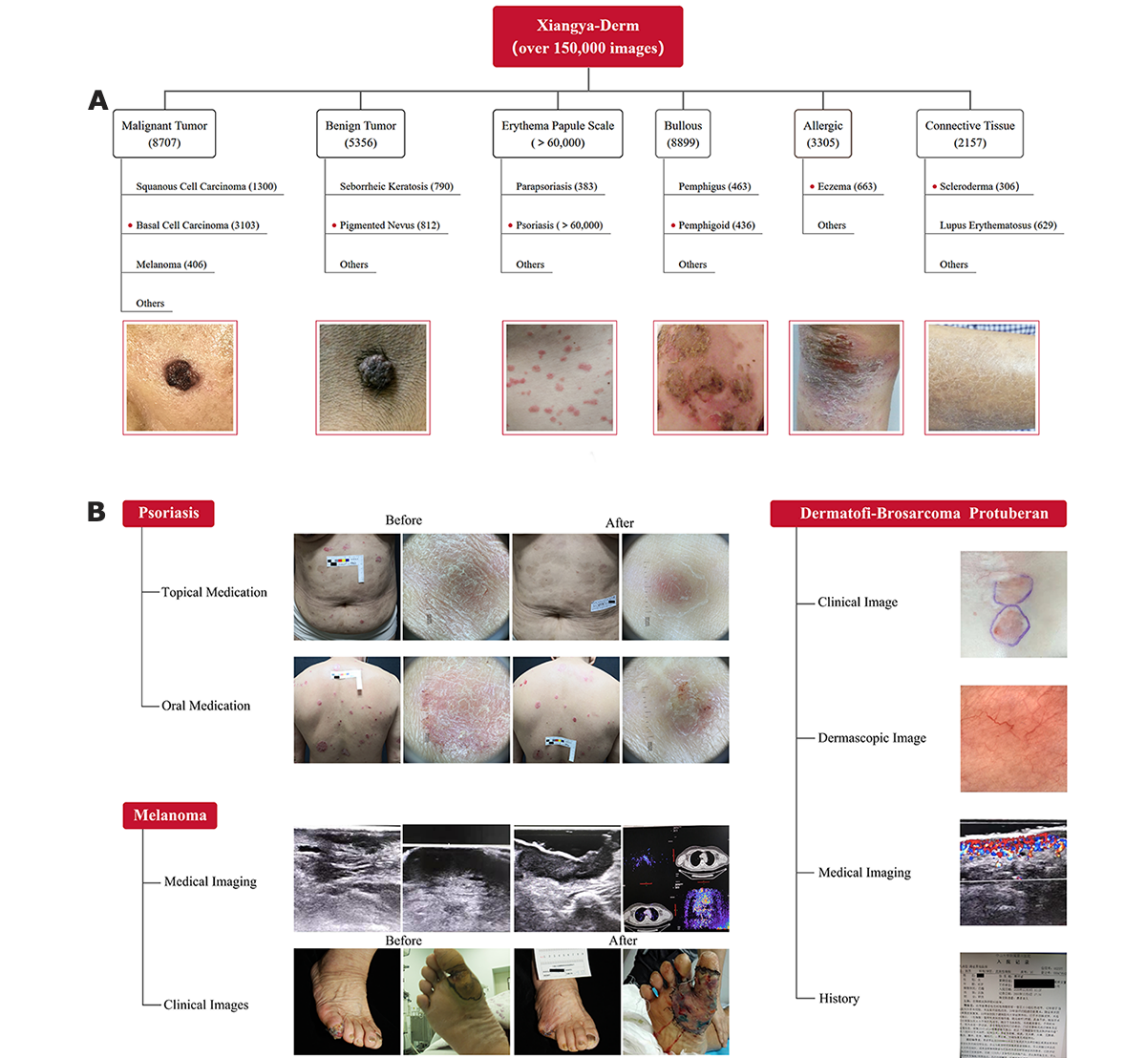
Branching tree diagram of the Xiangya-Derm database.

There are over 150,000 images in the Xiangya-Derm database, including 571 different skin diseases. Approximately, 60,000 images have been assigned annotations of skin type and lesion location. Common skin diseases, such as benign and malignant skin tumors, erythema papule scales, allergic skin diseases, and bullous skin diseases, are included (Figure 3). Of the total images in the database, 14,063 images are of skin tumors, including 5356 images of malignant tumors and 8707 images of benign tumors.

For common skin diseases, the diagnosis was independently made by three dermatologists based on history, clinical images, and dermoscopic features (Figure 2A). For rare skin diseases and skin tumors, the diagnosis was made based on history, clinical images, dermoscopic features, and histopathological features (Figure 2B). All the information about the patients and their diseases was collected in our database.

The database mainly consists of six types of skin diseases: malignant tumor, benign tumor, erythema papule scale, connective tissues, allergic, and bullous (Figure 3A). Representative examples in the Xiangya-Derm database are presented in Figure 3B, including psoriasis, melanoma, and dermatofibrosarcoma protuberans (an extremely rare disease). In addition to clinical images, dermascopic images, medical imaging information (eg, B-scan ultrasonography, positron emission tomography–computed tomography [PET-CT]) and history were included.

### Xy-SkinNet Performance on Six Common Skin Diseases

The performance when using different CNN structures as the backbone model on the testing data set is shown in Table 1. The ResNet-152 model showed better performance than others in terms of top 1 and top 3 mean accuracies (61.48% and 77.38%, respectively). Xy-SkinNet’s top differential diagnosis was for pemphigus and had a top 1 accuracy of 80.0%. When Xy-SkinNet was allowed three diagnoses, its top 3 accuracy rose to 90.0%. We also found that the performance of the ResNet-101 model was similar to that of the ResNet-152 model for certain diseases, such as seborrheic keratosis, pemphigus, and lupus erythematosus. However, the ResNet-101 model was better than the ResNet-152 model in terms of the top 1 accuracy on eczema (59.1% vs 54.5%) and the top 3 accuracies on lupus erythematosus (70.0% vs 60.0%).

**Figure 4:**
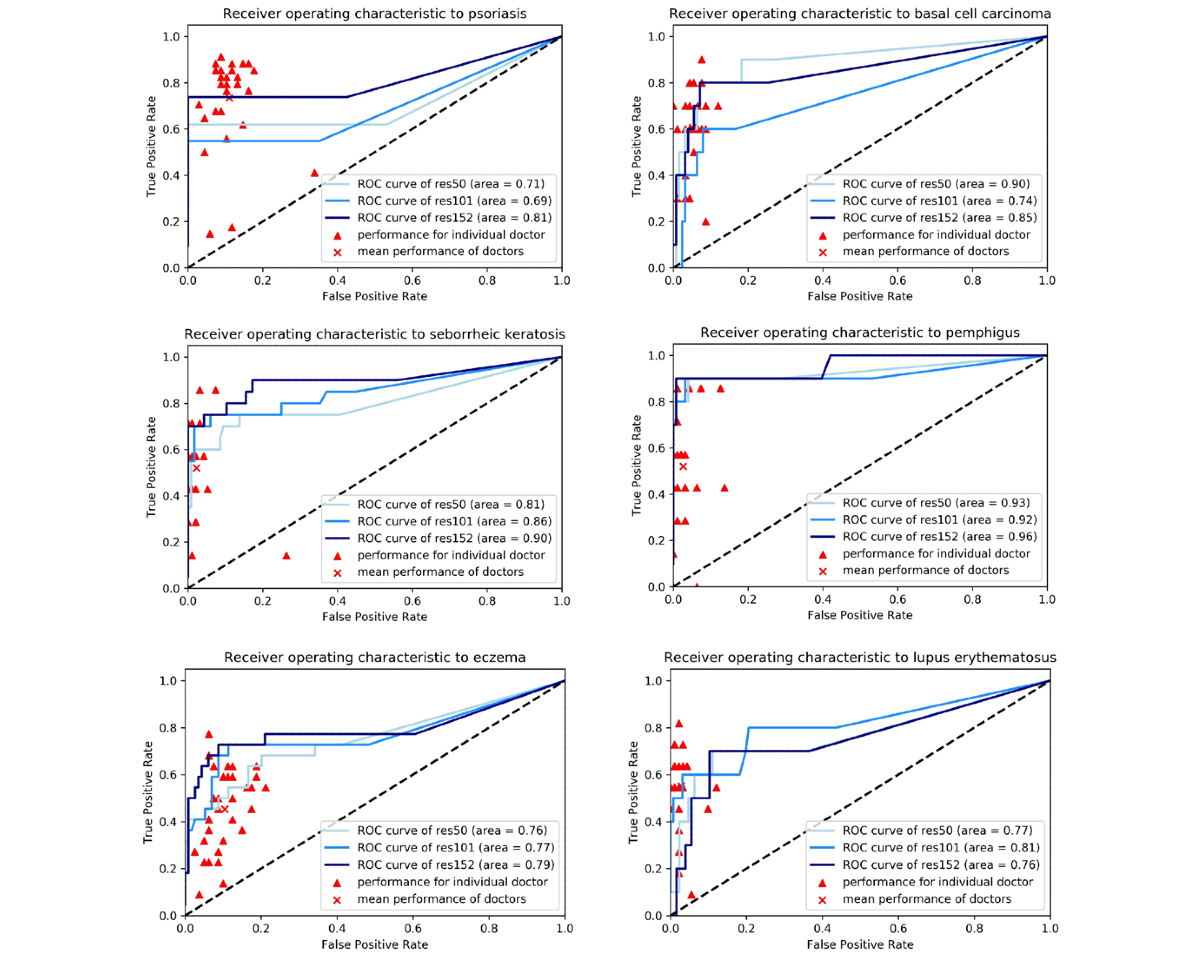
ROC curve of the six-category classification model. The three blue lines represent the mean ROC curves over the ResNet50, ResNet101, and ResNet152 runs. Each triangular point represents the results of one dermatologist. Results suggested that the mean performance of the dermatologists was lower than that of the models for most diseases. ROC: receiver operating characteristic.

The comparison between the experimental results of Xy-SkinNet and the average performance of dermatologists is shown in Table 2. The receiver operating characteristic (ROC) curve of the model for six types of diseases is shown in Figure 4, in which the performance of each dermatologist is also plotted. As the results showed (Table 2), Xy-SkinNet achieved a top 1 accuracy of 64.75%, while the average performance of the dermatologists was 62.13%. Moreover, Xy-SkinNet outperformed the dermatologists in terms of the top 3 accuracy, where Xy-SkinNet achieved an accuracy of 84.77%, while the dermatologists achieved an accuracy of 78.15%. As shown in Figure 4, for Xy-SkinNet, the area under the curve (AUC) values for the six types of diseases were 0.81, 0.85, 0.90, 0.96, 0.79, and 0.76, respectively, which were higher than the corresponding values of the average performance level of the dermatologists.

Moreover, we recorded the time costs for Xy-SkinNet and each dermatologist in our experiments. The results showed that Xy-SkinNet takes only 10 seconds to identify all 100 clinical images, while some dermatologists took over 2500 seconds or over 25 seconds for a single image. Even dermatologists with minimal time costs took up to 1216 seconds to classify the images. These results showed that the method adopted in this paper can achieve comparable performance in terms of accuracy to that of dermatologists and outperform dermatologists in terms of time costs.

The comparison of accuracies (%) on six different skin diseases between Xy-SkinNet and the average of dermatologists with different levels in the external data set provided by dermatologists are shown in Table 2. Xy-SkinNet achieved a top 1 accuracy of 64.75%, while the average performance of the dermatologists was 62.13%. Moreover, Xy-SkinNet outperformed the dermatologists in terms of the top 3 accuracy, where Xy-SkinNet obtained an accuracy of 84.77%, while the average performance of the dermatologists was 78.15%.

**Table 1:** Classification accuracies (%) of Xy-SkinNet.^a^

Backbone		PSO^b^ (%)	BCC^c^ (%)	SEK^d^ (%)	PEM^e^ (%)	ECZ^f^ (%)	LUE^g^ (%)	Mean (%)
**Top 1**								
	ResNet-50	61.9	20.0	60.0	70.0	45.5	40.0	49.57
	ResNet-101	54.8	20.0	70.0	80.0	59.1	50.0	55.65
	ResNet-152	69.4	40.0	75.0	80.0	54.5	50.0	61.48
	Xy-SkinNet	64.1	50.0	70.0	75.0	83.9	45.5	64.75
**Top 3**								
	ResNet-50	66.7	70.0	75.0	90.0	68.2	60.0	71.65
	ResNet-101	61.9	40.0	80.0	90.0	72.7	70.0	69.10
	ResNet-152	71.6	80.0	90.0	90.0	72.7	60.0	77.38
	Xy-SkinNet	67.7	70.0	90.0	90.0	100.0	90.9	84.77

^a^The classification accuracies (%) of Xy-SkinNet on six different skin diseases using different backbone networks as the feature extractor are shown. The ResNet-152 model showed better performance than the ResNet-50 and ResNet-101 models in terms of top 1 and top 3 mean accuracies. However, our original algorithm performed better than the others, especially in terms of the top 3 accuracies.

^b^PSO: psoriasis.

^c^BCC: basal cell carcinoma.

^d^SEK: seborrheic keratosis.

^e^PEM: pemphigus.

^f^ECZ: eczema.

^g^LUE: lupus erythematosus.

**Table 2:** Comparison of accuracy (%) between Xy-SkinNet and the average of dermatologists.

Class	Sample size	Top 1 (computer; %)	Top 1 (dermatologists; %)	Top 3 (computer; %)	Top 3 (dermatologists; %)
LUE^a^	11	45.5	60.6	90.9	70.0
ECZ^b^	31	83.9	47.5	100.0	76.9
SEK^c^	10	70.0	60.8	90.0	77.9
PSO^d^	93	64.1	75.7	67.7	87.2
BCC^e^	10	50.0	67.4	70.0	84.9
PEM^f^	20	75.0	60.8	90.0	72.0
All	175	64.75	62.13	84.77	78.15

^a^LUE: lupus erythematosus.

^b^ECZ: eczema.

^c^SEK: seborrheic keratosis.

^d^PSO: psoriasis.

^e^BCC: basal cell carcinoma.

^f^PEM: pemphigus.

## Discussion

### Principal Results

In this study, we established a new database, Xiangya-Derm, which consists of over 150,000 clinical images of 571 different skin diseases in the Chinese population. Xiang-Derm is the first integrated, normative database based on skin conditions in the Chinese population. Based on this database, we selected six common skin diseases and proposed an AI network, Xy-SkinNet. The top 1 and top 3 diagnostic accuracies of Xy-SkinNet were higher than those of dermatologists from the Department of Dermatology. This study was an attempt at exploring AI products and services and has successfully set the stage for future development. An increasing number of studies are incorporating clinical images [18-21].

There are already some open databases, such as AtlasDerm, Derm101, and Dermnet. Considering differences in skin color, Xiangya-Derm can provide data for realizing AI diagnosis of skin diseases among the Chinese population. Many existing databases lack medical history information, especially information about pathological diagnosis, and, potentially, contain some misdiagnosed photos. Notably, one of the greatest advantages of Xiangya-Derm is that most images contain corresponding skin pathology results, providing the category annotation of a gold standard, which can be most effectively applied to various research studies and in the development of AI. This feature ensures that the diagnostic information about pictures used for deep learning is accurate and reduces the diagnostic errors caused by misdiagnosis. Of course, there are also a small number of unmatched pictures in our database, which is correlated with the lack of corresponding dermoscopic images.

In addition, XiangyaDerm provides image data with the location for all skin lesions, thus enabling researchers to apply object detection algorithms in computer vision for the automatic diagnosis of skin diseases. Moreover, each image has a full set of clinical information about the patient, including demographic information, complaints, current medical history, past medical history, and family history. Given the complete set of big data, conducting further research on AI diagnosis using multimodal data, which is more coincident with the real-world diagnosis process and more intuitive for both doctors and patients, is achievable.

### Comparison With Prior Work

Many attempts have been made in the field of automatic diagnosis of dermatosis. For example, Alćonet et al [22] proposed a system that consisted of a feature extractor and a classifier. This system is capable of adapting its decision-making process based on patient information. Blum et al [23] performed digital image analysis using 64 different analytical parameters based on 837 cases of melanocyte lesions to establish a computer algorithm for diagnosing melanocyte lesions. The diagnostic accuracies of the algorithm for complete and partial imaging were 82% and 84%, respectively. Another study used multiple databases to perform dermoscopic and digital dermoscopic examinations of cutaneous melanoma and compared the diagnostic accuracies of different dermal algorithms and digital dermatoscopy joint intelligence [24].

CNNs have presented varying results in medical fields when using an improved algorithm [8,25,26]. Pal et al [10] designed and produced a psoriasis skin biopsy image data set of 90 images and attempted to segment the images using several different neural network models. Their results were superior to the manual extraction features. Sun et al [9] attempted to classify dermatological images using CNNs as feature extraction methods. They used a data set that included 198 types of diseases and 6584 images. However, despite using a large data set, the final result was inferior to that obtained by the use of AI extraction features. Han et al [27] used a deep learning algorithm to classify clinical images of 12 dermatoses, with performance comparable to that of 16 dermatologists. Tschandl et al [28] showed that computers can achieve an accuracy similar to that of medical students by using a CNN-based method to diagnose skin diseases on an image. In our study, the AI-based six-category classification outperformed the dermatologists. Patients’ multimodal data were used to create a simulated clinical setting for AI. Thus, the comparison between AI and the dermatologists is fair as the diagnosis made by dermatologists in real clinical practice partially depends on history and other nonimage information.

Relevant research reports also increasingly support that deep learning systems can help improve the accuracy of nondermatologists or clinicians with a lack of experience, especially in primary care [1]. AI can also foster and improve the confidence of doctors. In China, 95% of dermatologists are concentrated in tertiary hospitals, and the number of dermatologists at county hospitals, especially township hospitals, is less than 5% of the total number. In addition, numerous county and township hospitals have a limited number of dermatologists. The proportion of doctors and patients is unbalanced, and the level of doctors is uneven, causing delayed diagnosis for many patients, poor prognosis, aggravation of the family economic burden, etc [29]. In our research, we selected six skin diseases: psoriasis, basal cell carcinoma, seborrheic keratosis, pemphigus, eczema, and lupus erythematosus. Our research may satisfy the needs of primary doctors, as these six conditions are common in primary care and outpatient work. In the future, we intend to explore an auxiliary diagnosis system that can provide dermatologists with three possible diagnoses, ranked by accuracy, for one lesion. The risk of misdiagnosis is likely to decline for primary diagnoses when combining AI and manual correction.

Because there are few dermatologists in China’s rural areas, we plan to build a multidimensional AI platform that assists Chinese primary dermatologists in diagnosing skin diseases through deep learning and the construction of Xiangya-Derm. In light of the lack of criteria for skin disease data sets, we would like to encourage the establishment of standards in the future. It would be beneficial to create a new data set or improve the existing data sets with multimodal clinical information.

### Limitations

Although we included six types of skin diseases in our data set, a large number of skin diseases were not considered. One of the reasons for this is that these six diseases cover most of the skin conditions in clinical practice. With this database and the development of AI products, new data for rare skin diseases can be collected at the same time as the diagnosis of common skin diseases. In addition, the quality of health services has improved in China, and some skin diseases, such as bacterial and parasitic skin diseases, are rarely seen. Thus, it is difficult to collect data on these diseases. Moreover, the clinical images in our database were collected from the Chinese population, making the database ineffective for training an intelligent global skin disease diagnosis system.

### Conclusions

Xiangya-Derm, the largest database for skin diseases in the Chinese population, was created, and six common skin conditions were classified based on Xiangya-Derm to lay a foundation for product research.

## Abbreviations

AI: artificial intelligence
AUC: area under the curve
CNN: convolutional neural network
EU: European Union
M-ResNet: modified ResNet
PET-CT: positron emission tomography–computed tomography
ROC: receiver operating characteristic
RPN: region proposal network
